# High-Power Laser Therapy Modulates Mitochondrial Function and Redox Balance Without Cytotoxicity: An In Vitro Study in BV-2 Microglial Cells

**DOI:** 10.3390/antiox14101243

**Published:** 2025-10-16

**Authors:** Luana Barbosa Dias, Thiago De Marchi, Ana Paula Vargas Visentin, Juliana Maria Chaves, Catia Santos Branco, Fernando Joel Scariot, Matheus Marinho Aguiar Lino, Older Manoel Araújo-Silva, Amanda Lima Pereira, Heliodora Leão Casalechi, Douglas Scott Johnson, Shaiane Silva Tomazoni, Ernesto Cesar Pinto Leal-Junior

**Affiliations:** 1 Laboratory of Phototherapy and Innovative Technologies in Health (LaPIT), Postgraduate Program in Rehabilitation Sciences, Nove de Julho University, São Paulo 01504-001, SP, Brazil; 2 Laboratory of Health, Innovation and Therapeutic Technologies (LabHITT), Center of Excellence in Entrepreneurship and Innovation (CE^2^I), Uniftec University Center, Caxias do Sul 95012-669, RS, Brazil; 3 Laboratory of Oxidative Stress and Antioxidants, Institute of Biotechnology, University of Caxias do Sul, Caxias do Sul 95070-560, RS, Brazil; 4 Multi Radiance Medical, Solon, OH 44139, USA; 5 ELJ Consultancy–Scientific Consulting, São Paulo 01153-000, SP, Brazil

**Keywords:** high-intensity laser, oxidative stress, mitochondrial function, safety profile

## Abstract

Background: Recent technological advances have sparked growing interest in high-power laser devices due to their capacity for energy delivery and therapeutic potential, especially in deeper tissues. This promising approach may be comparable to photobiomodulation for modulating inflammatory and redox processes in various tissues. However, to our knowledge, this is the first study to evaluate the safety profile and redox modulation capacity of high-power laser therapy in BV-2 microglial cells. Methods: This study investigated the cellular responses of BV-2 microglial cells exposed to three laser irradiation protocols using a high-power laser device (650/810/915/980 nm, 657 J total dose), applied at variable distances to simulate in vivo power attenuation. Cell viability, apoptosis, adenosine triphosphate(ATP) levels, mitochondrial membrane potential (MMP), reactive oxygen species (ROS), nitric oxide (NO), and intracellular calcium levels were assessed at multiple time points (5 min to 24 h). Results: Protocol-dependent effects were observed. Protocol A promoted early increases in cell viability and ATP levels, along with decreased apoptotic markers and ROS production, suggesting a protective bioenergetic response. In contrast, Protocol C showed transient increases in oxidative stress and reduced MMP, suggesting possible mitochondrial stress. A selective increase in NO levels under Protocol A also suggests modulation of inflammatory pathways without cytotoxicity. Conclusions: High-power laser therapy modulates redox balance, mitochondrial function, and inflammatory mediators (e.g., NO) in a dual-phase manner in BV-2 microglial cells. These findings contribute to defining safe and effective parameters for potential musculoskeletal and neurological applications.

## 1. Introduction

Photobiomodulation (PBM) is a non-invasive therapeutic approach that uses light to stimulate biological processes and promote tissue recovery. Clinically, PBM has been applied in the treatment of pain, wound healing, tissue repair (including nerves, muscles, and tendons), modulation of inflammatory responses, and reduction in muscle fatigue, with generally favorable outcomes. To date, reported adverse effects have mainly been associated with accidental retinal exposure [1]. Most studies have focused on low-power lasers (≤500 mW), which deliver non-thermal energy and induce photochemical effects that depend on parameters such as wavelength, irradiation mode (continuous or pulsed), fluence, output power, and irradiance. When using pulsed modes, additional factors such as pulse duration and interval must also be considered. In contrast, recent advances in high-power laser devices (>500 mW, class IV) have increased interest in their clinical use due to their ability to deliver higher doses of energy to deeper tissues in shorter times. However, this enhanced penetration raises both therapeutic potential and possible risks, such as tissue heating, making it essential to investigate their safety profile and confirm their biological effects [2].

Microglial cells, widely used in vitro through the BV-2 cell line, are the primary immune effector cells of the central nervous system (CNS). They act as sentinels, continuously surveying the CNS microenvironment, remodeling synapses, and rapidly responding to pathological stimuli [3]. Microglia play a pivotal role in neuroinflammation, oxidative stress responses, and the regulation of neuronal survival. Under pathological conditions, their activation can lead to the release of reactive oxygen species (ROS), pro-inflammatory cytokines, and other mediators that influence neuronal function and contribute to disease progression [4]. Given their central role in inflammatory and oxidative processes within the CNS, BV-2 cells represent an appropriate model for investigating how external stimuli may impact microglial physiology. Activation of microglia is accompanied by the release of several mediators, including pro-inflammatory cytokines and ROS, which can impair neuronal function and exacerbate neuroinflammatory processes [5,6,7]. These processes are therefore critical to consider when evaluating potential cytotoxic or modulatory effects in experimental models.

Several studies have suggested that PBM may modulate cellular behavior by modifying key functions such as cytokine release, phagocytic activity, redox balance, and mitochondrial bioenergetics [8]. These effects appear to occur within a therapeutic window of action influenced not only by the dose but also by parameters such as wavelength, fluence (energy density), and irradiance (power density) [1]. While most of this evidence comes from studies using low-power lasers, the question remains whether high-power laser therapy (class IV) elicits similar or distinct cellular responses. Due to its higher energy delivery and deeper tissue penetration, class IV laser therapy could enhance or even modify the modulatory mechanisms described for PBM, but it may also introduce additional risks associated with the high power, speed, and magnitude of energy delivery to the cells. Understanding whether high-power lasers reproduce the beneficial, non-cytotoxic effects of PBM or whether they trigger different biological outcomes is therefore critical to establishing their safety and therapeutic potential.

Therefore, this study aimed to evaluate the safety profile of class IV laser therapy in BV-2 microglial cells, focusing on cell viability, oxidative stress, and mitochondrial function, in association with apoptosis and inflammation.

## 2. Materials and Methods

### 2.1. Reagents

Fetal bovine serum (FBS) was obtained from Laborclin (Pinhais, PR, Brazil). Penicillin-streptomycin, 3-(4,5-dimethylthiazol-2-yl)-2,5-diphenyltetrazolium bromide (MTT), RPMI-1640 medium, 3,3′-dihexyloxacarbocyanine iodide, 2′,7′-dichlorofluorescin diacetate, and trypsin-EDTA were supplied by Sigma-Aldrich (St. Louis, MO, USA). The PicoGreen™ assay and the Annexin V-FITC/propidium iodide apoptosis detection kit were provided by Thermo Fisher Scientific (São Paulo, SP, Brazil). The mitochondrial potential probe DioC_6_(3) was purchased from Molecular Probes (Eugene, OR, USA). ATP content was measured using the CellTiter-Glo^®^ luminescent assay (Promega, Madison, WI, USA). All additional chemicals and solvents used were of analytical grade and purchased from Sigma.

### 2.2. Cell Culture

BV-2 mouse microglial cells (BCRJ Cat# 0356, Rio de Janeiro Cell Bank, Brazil) were used in this study. BV-2 cells are widely recognized as an in vitro model for microglial activity, due to their immunological functions in the central nervous system (CNS), including roles in neuroinflammation, synaptic remodeling, and neuronal survival [9,10]. These characteristics justify their selection for evaluating cellular responses to high-power laser therapy. The cells were cultured in RPMI 1640 medium supplemented with 10 mM HEPES, 10% fetal bovine serum (FBS), and 1% penicillin (100 U/mL)–streptomycin (100 μg/mL). Cultures were maintained in a humidified incubator with 5% CO_2_ at 37 °C under standard conditions. Experiments were conducted when cells reached 80–90% confluence.

### 2.3. Laser Treatment

To account for the attenuation of power and energy by skin layers under in vivo conditions, our research group conducted a preliminary experiment to quantify the high-power laser’s output after it passed through a tissue equivalent. Based on those findings, we developed a standardized in vitro application model using a scanning technique over culture plates. This model maintains a fixed distance between the laser emitter and the cell surface for each irradiation protocol, thereby mimicking the power and energy attenuation observed in a live biological system. While the total delivered energy was held constant across protocols, irradiance (power density) and exposure time were intentionally varied (Table 1) to model how the rate of energy delivery, a key determinant of thermal and photochemical dynamics, can modulate cellular responses despite identical total energy.

For cell treatment, a high-power laser device (Alpha^®^, Multi Radiance Medical^®^, Solon, OH, USA) was used, featuring adjustable wavelengths (650, 810, 915, and 980 nm), variable power output ranging from 200 to 14,400 mW, and different probes with aperture areas of 1, 5, and 20 cm^2^. Since the application was performed directly onto plates, we used the 5 cm^2^ probe. The total energy dose applied to all groups was 657 J, a parameter recommended by the manufacturer and described in the device’s treatment protocols.

The treatments were carried out using three distinct experimental protocols (Protocol A, Protocol B, and Protocol C). For each protocol, a specific attenuation distance was employed, determined by measurements using a laser distance meter: 34.0 cm for Protocol A, 33.0 cm for Protocol B, and 33.1 cm for Protocol C. The complete set of parameters used in each protocol is detailed in Table 1.

### 2.4. Experimental Timeline and Controls

BV-2 cells were irradiated and assayed at 5 min, 30 min, 1 h, 3 h, and 24 h post-irradiation, as indicated in the figures. For each time point and protocol (A–C), as well as for the non-irradiated control, independent wells from the same seeding batch were used; no well was measured more than once. All assays employed endpoint or destructive procedures (e.g., CellTiter-Glo^®^ for ATP, DCFH-DA for ROS, DiOC_6_(3) for MMP, Fluo-3 for Ca^2+^, Annexin-V/PI for apoptosis), which did not allow repeated measurements in the same well.

The control condition consisted of non-irradiated BV-2 cells maintained under identical culture conditions. A separate sham-irradiation group was not included, as the non-irradiated control adequately represented basal cell responses under the experimental setup.

### 2.5. Replicates, Randomization, and Blinding

Each condition at each time point was tested in three independent biological experiments (n = 3), conducted on different days, with technical triplicates in each experiment. Wells were randomly assigned to groups. Data acquisition (plate reader and flow cytometry—10,000 events/sample) was carried out by trained personnel, and quantitative analyses were conducted by investigators blinded to protocol allocation, using FlowJo (for cytometry) and the instrument’s analysis software for plate-based assays.

### 2.6. Cell Viability

Cell viability was evaluated by the MTT [3-(4,5-dimethylthiazol-2-yl)-2,5-diphenyltetrazolium bromide] method. In brief, 1 × 10^5^ cells were seeded into 96-well plates and allowed to attach for 24 h. After exposure to high-power laser treatment, 100 µL of MTT solution (1 mg/mL in RPMI-1640 medium without supplements) was added to each well, followed by a 2 h incubation at 37 °C. The medium was then discarded, and the formazan crystals formed were dissolved in 300 µL of dimethyl sulfoxide (DMSO) with gentle mixing at room temperature. Absorbance was subsequently recorded at 517 nm using a Victor-X3 multimode microplate reader (PerkinElmer, Turku, Finland). Data are expressed as relative absorbance values [5].

### 2.7. ATP Production

Intracellular ATP levels were assessed using the CellTiter-Glo^®^ Luminescent Cell Viability Assay (Promega, Madison, WI, USA) in accordance with the manufacturer’s guidelines. BV-2 cells (5 × 10^4^ per well) were seeded into opaque 96-well plates, allowed to grow for 24 h, and subsequently exposed to high-power laser treatment. Luminescence was recorded using a multi-mode microplate reader (Victor-X3, Perkin Elmer, Turku, Finland). The results of the ATP measurements were expressed as relative light units (RLU) [5].

### 2.8. Flow Cytometry

Cells were grown in 75 cm^2^ flasks (9 × 10^5^ cells/mL). For all cytometry assays, irradiated and non-irradiated controls were detached using trypsin-EDTA, washed with PBS, and processed in parallel under identical conditions.

Flow cytometry was used to quantify intracellular reactive oxygen species (ROS), mitochondrial membrane potential (MMP), apoptosis, and intracellular calcium levels, following established protocols by Frozza et al. [9] and Sekar et al. [10].

Following treatments and cell preparation, fluorescence from 10,000 cells was recorded using a four-color BD FACSCalibur flow cytometer (Becton Dickinson, São Paulo, Brazil). Data acquisition was performed with CellQuest Pro software, version 5.1 (BD Biosciences, Franklin Lakes, NJ, USA), and data analysis was conducted using FlowJo software, version 10 (TreeStar, Inc., Ashland, OR, USA). Fluorescence signals were background-subtracted and expressed as arbitrary fluorescence units (AFU). For graphical presentation, values were normalized to the mean of the time-matched non-irradiated control (set at 100%) and are reported as % of control. Raw AFU values are provided in Supplementary Table S1.

### 2.9. Intracellular Calcium Levels

For intracellular calcium analysis, cells were incubated with 1 μM Fluo-3 AM (Calcium Indicator, Thermo Fisher Scientific, Waltham, MA, USA) for 30 min at 37 °C in the dark. Cells were then washed twice with PBS and resuspended in 500 μL of PBS. Intracellular calcium levels were expressed as relative fluorescence intensity.

### 2.10. Intracellular Reactive Oxygen Species

Reactive oxygen species (ROS) production was assessed using 2′,7′-dichlorodihydrofluorescein diacetate (DCFH-DA; Sigma-Aldrich, St. Louis, MO, USA). Once inside the cells, DCFH-DA is deacetylated and subsequently oxidized by ROS, yielding the fluorescent product dichlorofluorescein (DCF). Cultures were treated with a 10 µM DCFH-DA solution and incubated for 30 min at 37 °C in the dark. Fluorescence signals were then recorded and reported as relative fluorescence units (RFU), reflecting intracellular ROS levels for each experimental group [5].

### 2.11. Mitochondrial Membrane Potential

Alterations in mitochondrial membrane potential (MMP) were determined using the fluorescent dye 3,3′-dihexyloxacarbocyanine iodide [DiOC_6_(3)]. Cells were incubated with DiOC_6_(3) (175 nM) for 30 min at room temperature, shielded from light. After staining, fluorescence was measured through the FL1 channel of a flow cytometer. Data were expressed as relative fluorescence units (RFU), representing variations in mitochondrial polarization [5].

### 2.12. Annexin-V/PI Assay (Apoptosis)

Apoptotic cells were identified using the Annexin V-FITC/propidium iodide (PI) assay kit (Thermo Fisher, São Paulo, Brazil). Cell pellets were resuspended in PBS (pH 7.4) and mixed with 100 µL of binding buffer (10 mM HEPES/NaOH, pH 7.4; 140 mM NaCl; 2.5 mM CaCl_2_). Subsequently, 5 µL of Annexin V-FITC and 10 µL of PI were added to each sample, followed by a 15 min incubation at room temperature, protected from light. Fluorescence was analyzed on a flow cytometer using FL1 (488/533 nm) and FL3 (488/670 nm) channels. Results were reported as the percentage of positive cells [5].

### 2.13. Extracellular DNA Measurement

Quantification of extracellular double-stranded DNA (dsDNA) was performed using the Quant-iT™ PicoGreen assay (Thermo Fisher Scientific, Waltham, MA, USA), a dye highly selective for dsDNA. Following high-power laser treatment, 10 µL of culture supernatant was transferred to a black 96-well plate containing 80 µL of 1× TE buffer. Each well then received 10 µL of PicoGreen working solution and was incubated for 5 min at room temperature in the dark. Fluorescence was measured at 480 nm excitation and 520 nm emission. Data were expressed as relative fluorescence units (RFU), indicating extracellular dsDNA content for each experimental condition [5].

### 2.14. Nitric Oxide Measurement

Nitric oxide (NO) production was assessed indirectly through the Griess reaction, which measures nitrite and nitrate as end-products of NO metabolism. After laser exposure, 50 µL of supernatant was transferred to a new 96-well plate and combined with an equal volume of Griess reagent (1:1, *v*/*v*). The mixture was incubated for 10 min at room temperature in the dark, and absorbance was read at 550 nm using a Victor-X3 microplate reader (PerkinElmer, Turku, Finland). Results were reported as relative absorbance values, corresponding to extracellular NO concentrations [5].

### 2.15. Statistical Analysis

Data are expressed as mean ± SD. Normality was assessed before conducting inferential tests. Given the factorial, between-samples design, outcomes were analyzed using two-way ANOVA (factors: Protocol and Time), followed by Tukey’s post hoc multiple comparisons test. Biological replicates (n) refer to independent experiments performed on different days, with technical triplicates averaged within each experiment. Statistical significance was set at *p* < 0.05.

## 3. Results

The measurements of all variables analyzed are presented in Table S1 (Supplementary Materials).

### 3.1. Cell Viability

**Figure 1:**
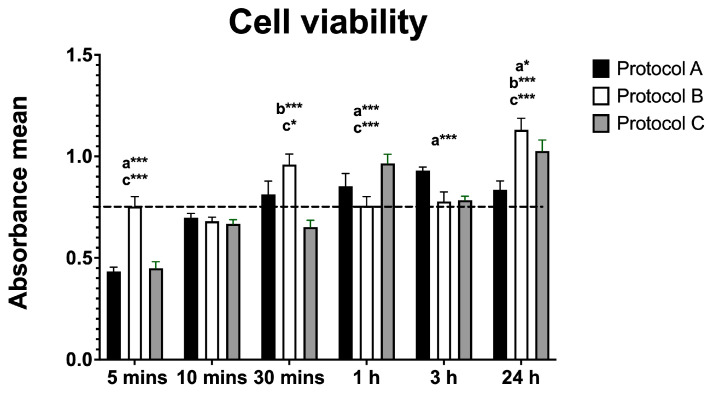
Data are presented as mean ± SD. The dotted horizontal line represents the mean value of the non-irradiated control. Small letters indicate protocol type. * *p* < 0.05, *** *p* < 0.001.

Cell viability analysis (based on mean absorbance values) revealed significant changes across all protocols compared with the control. Protocol A showed an initial decrease in viability at 5 min, followed by significant increases at 1 h and 24 h. Protocol B induced a significant increase at 30 min and again at 24 h, with no significant differences at 1 or 3 h. Protocol C showed an initial decrease in viability at 5 and 30 min, followed by significant increases at 1 h and 24 h, along with a transient decline at 3 h. These results indicate a biphasic effect of irradiation, with early reductions and later enhancements in cell viability, particularly under Protocol C (Figure 1).

### 3.2. ATP Production

**Figure 2:**
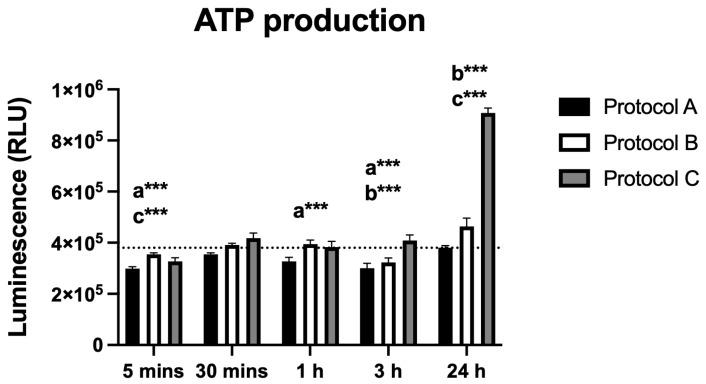
Data are presented as mean ± SD. The dotted horizontal line represents the mean value of the non-irradiated control. Small letters indicate protocol type. *** *p* < 0.001.

Irradiation led to significant changes in ATP production, as measured by luminescence (RLU). Protocol A caused decreased ATP production at most time points, with values comparable to the control only at 30 min and 24 h. Protocol B showed a significant reduction at 3 h, followed by a marked increase at 24 h. Similarly, Protocol C produced a significant rise in ATP levels at 24 h. These findings indicate that ATP production is modulated by both the timing and the irradiation protocol used (Figure 2).

### 3.3. Intracellular Calcium Levels

The analysis of intracellular calcium levels revealed time-dependent changes following irradiation with protocols A, B, and C. Protocol A induced a significant increase in calcium levels, peaking at 30 min post-irradiation, followed by a gradual decline up to 24 h. All time points showed statistically significant differences compared to the control group.

Similarly, Protocol B resulted in a significant rise in calcium levels at 5 min, followed by a gradual decrease. Statistically significant differences were observed at all time points except 24 h. Protocol C displayed a comparable pattern, with peak levels at 30 min and a subsequent decrease; statistical significance was maintained at all time points except 24 h.

**Figure 3:**
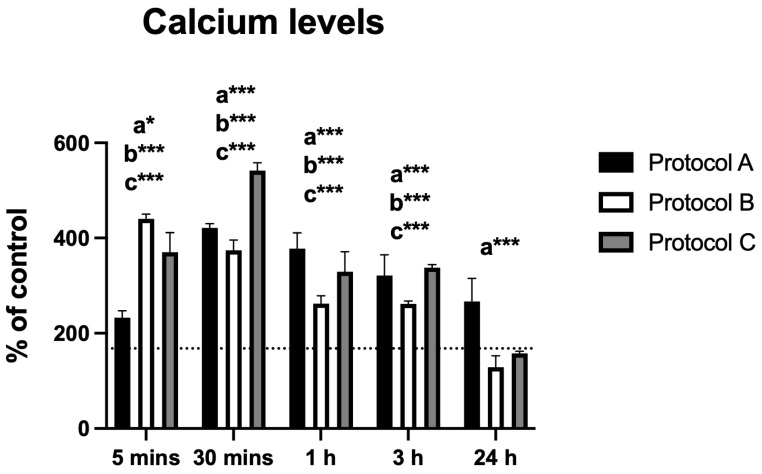
Data are presented as mean ± SD. The dotted horizontal line represents the mean value of the non-irradiated control. Small letters indicate protocol type. * *p* < 0.05, *** *p* < 0.001.

Overall, the protocols produced similar response trends, with the exception of Protocol B, which showed an earlier effect at 5 min without reaching a maximum at 30 min (Figure 3).

### 3.4. Reactive Oxygen Species (ROS)

**Figure 4:**
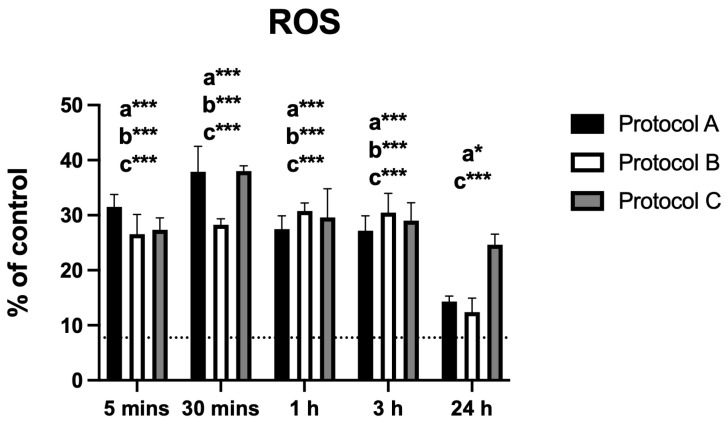
Data are presented as mean ± SD. The dotted horizontal line represents the mean value of the non-irradiated control. Small letters indicate protocol type. * *p* < 0.05, *** *p* < 0.001.

All irradiation protocols resulted in a significant increase in ROS production. Protocol A showed a marked rise within the first 30 min, followed by a progressive decline, although levels remained significantly elevated at 24 h. Protocol B induced increased ROS levels up to 3 h, after which they declined and were no longer statistically different from the control at 24 h. Protocol C peaked at 30 min and then decreased gradually but remained significantly higher than the control throughout the entire evaluation period (Figure 4).

### 3.5. Mitochondrial Membrane Potential (MMP)

**Figure 5:**
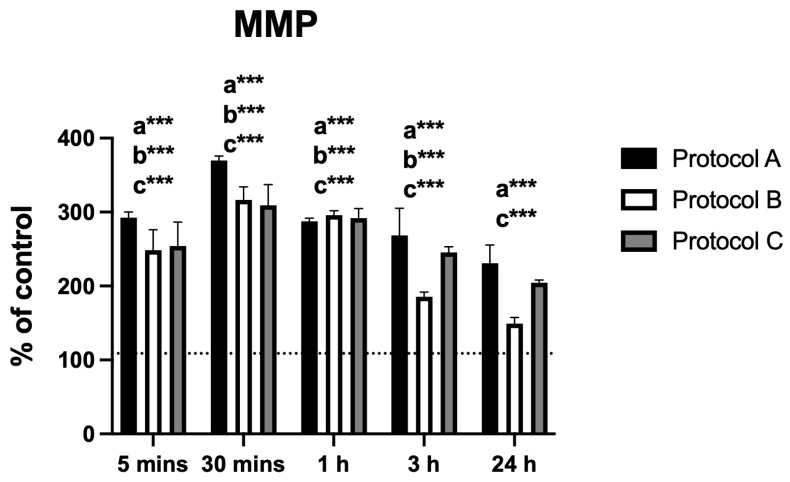
Data are presented as mean ± SD. The dotted horizontal line represents the mean value of the non-irradiated control. Small letters indicate protocol type. *** *p* < 0.001.

Changes in MMP displayed a pattern similar to that observed for intracellular calcium. Protocol A produced a marked increase in fluorescence intensity at 30 min, followed by a progressive decrease up to 24 h, with significant differences from the control at all time points. Protocol B showed a comparable trend, although the difference at 24 h was not statistically significant. Protocol C also peaked at 30 min and subsequently declined, while maintaining statistically significant differences from the control at all evaluated time points (Figure 5).

### 3.6. Apoptosis

**Figure 6:**
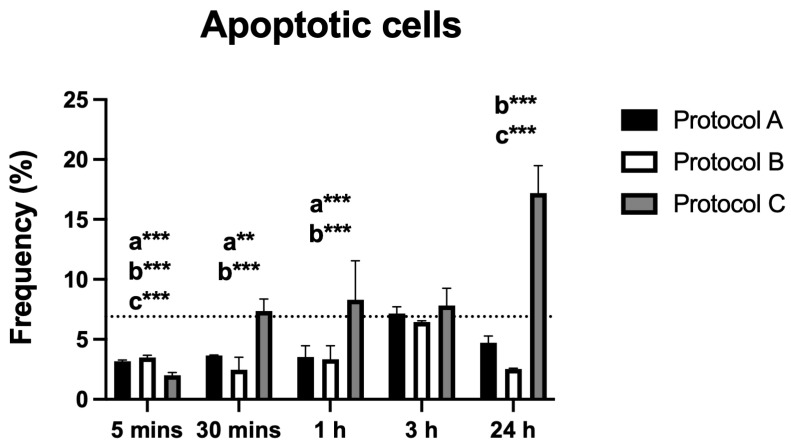
Data are presented as mean ± SD. The dotted horizontal line represents the mean value of the non-irradiated control. Small letters indicate protocol type. ** *p* < 0.01, *** *p* < 0.001.

Flow cytometric analysis demonstrated significant differences in the percentage of apoptotic cells across all irradiated groups compared to the control. Protocols A and B both showed a significant reduction in apoptosis that was sustained up to 3 h. Notably, Protocol B also exhibited a renewed statistically significant reduction at 24 h. In contrast, Protocol C displayed the opposite effect, with a significant increase in apoptotic cells observed at 24 h post-irradiation (Figure 6).

### 3.7. dsDNA Release

**Figure 7:**
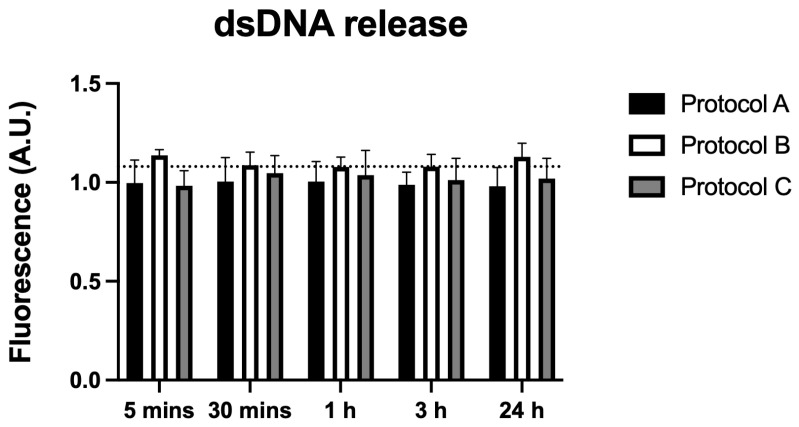
Data are presented as mean ± SD. The dotted horizontal line represents the mean value of the non-irradiated control. No significant differences were found when comparing the groups with the control.

Analysis of extracellular dsDNA levels revealed no statistically significant differences between any irradiated groups and the control across all time points (5 min to 24 h). These findings indicate that the irradiation protocols did not induce dsDNA release, suggesting minimal cellular damage (Figure 7).

### 3.8. Nitric Oxide (NO) Production

**Figure 8:**
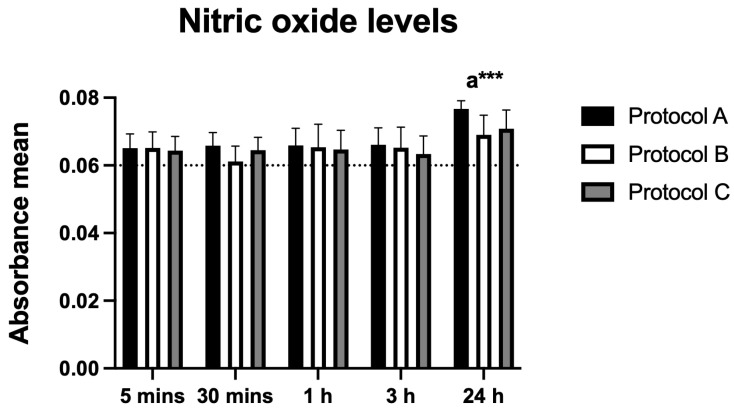
Data are presented as mean ± SD. The dotted horizontal line represents the mean value of the non-irradiated control. Small letters indicate protocol type. *** *p* < 0.001.

Nitric oxide (NO), assessed as an inflammatory marker, increased significantly under Protocol A at 24 h post-irradiation. Protocols B and C showed no significant changes at any time point. These findings suggest a specific modulatory effect of Protocol A on NO production in BV-2 cells (Figure 8).

## 4. Discussion

This study evaluated the safety profile, mitochondrial function, and potential mechanisms of action of high-power laser therapy in BV-2 microglial cells by examining key cellular parameters, including viability, ATP production, oxidative stress, mitochondrial membrane potential (MMP), apoptosis, and NO levels. Safety evaluation was primarily based on cell viability, apoptosis, and mitochondrial function (MMP and ATP). ROS and Ca^2+^ levels were analyzed as complementary indicators of redox homeostasis and intracellular signaling rather than as direct mitochondrial markers. The results revealed time- and protocol-dependent cellular responses, indicating that high-power laser therapy can modulate essential biological processes without inducing overt cytotoxicity. Reinforcing the novelty of our study, and based on searches across different databases, this is the first article to evaluate the application of high-power laser therapy on ATP production capacity (a direct marker of mitochondrial bioenergetics), intracellular calcium flux, ROS, MMP, NO, and dsDNA release in BV-2 cells. Previous studies using PBM in microglia have reported modulatory effects on inflammatory pathways: Vogel et al. [11] demonstrated that low-level PBM reduced Iba-1 expression and pro-inflammatory cytokines in an in vivo ischemic stroke model, while Chen et al. [12] showed that 1070 nm PBM polarized BV-2 microglia toward an anti-inflammatory phenotype (M2), reducing pro-inflammatory mediators and enhancing neuroprotective signaling. These reports, together with our findings, highlight the responsiveness of microglia to laser-based interventions across different energy scales and support their potential as modulatory targets in neuroinflammatory contexts.

Cell viability data demonstrated a biphasic response to irradiation. Protocols A and C induced an early transient reduction (at 5 min), followed by a significant increase at later time points (1 h and 24 h), particularly in Protocol C. This late increase may reflect adaptive responses to laser stimulation, such as activation of survival pathways or metabolic compensation. Protocol B was less variable, showing increases at 30 min and 24 h. These results are consistent with the dose-dependent mechanism of PBM, in which low or moderate doses induce beneficial effects, while excessive exposure may generate transient stress responses [13,14]. In our study, the observed differences appear to reflect variations in delivery rate (irradiance and exposure duration) rather than total energy, since total energy was constant across protocols. Our results corroborate previous studies [14] reporting that high-intensity laser irradiation did not reduce cell viability in C2C12 myoblasts regardless of the protocol used.

Although total energy was identical across protocols, irradiance and exposure duration differed (A: 584 mW/cm^2^/225 s; B: 973 mW/cm^2^/135 s; C: 1460 mW/cm^2^/90 s). The more pronounced 24 h ATP increase under Protocol C likely reflects a delivery-rate effect, whereby higher irradiance with shorter exposure produces stronger early MMP and transient ROS/Ca^2+^ signals, which are known to activate redox- and Ca^2+^-sensitive pathways and mitochondrial bioenergetics [15,16]. Such signaling can manifest as a delayed enhancement of oxidative phosphorylation, consistent with the time lag between early MMP/ROS/Ca^2+^ changes and the ATP rise at 24 h. The lack of dsDNA release and the predominantly anti-apoptotic profile in Protocols A and B support a non-injurious, adaptive mechanism underlying these bioenergetic effects. Finally, cytochrome-c-oxidase photoacceptance and NO photodissociation under higher irradiance may further facilitate electron transport [17], contributing to the greater ATP recovery observed in Protocol C. The apparent paradox of higher ATP alongside a late increase in apoptosis in Protocol C can be reconciled by cell-fate heterogeneity and delivery-rate effects. While a susceptible subpopulation likely crossed a stress threshold (consistent with stronger early ROS/Ca^2+^/MMP transients under higher irradiance), the majority of surviving cells engaged adaptive, energy-dependent programs, resulting in a net rise in ATP levels at 24 h. Notably, apoptosis is ATP-dependent, and the lack of dsDNA release argues against necrotic damage, supporting a regulated cell-death process in a fraction of cells rather than widespread injury [16]. This heterogeneous, hormetic-like response is consistent with PBM literature, where delivery rate (irradiance × time), even under identical total energy, can drive divergent cell-fate trajectories [1,13]. Protocol A was associated with decreased ATP synthesis at several time points. The reduced ATP in microglial cells may directly affect their excitability and activity, resulting in lower release of pro-nociceptive mediators and, consequently, inhibition of pain conduction [14,18], which represents an interesting potential for in vivo applications.

Intracellular calcium levels were also modulated by laser treatment, with all protocols showing an initial peak (especially at 30 min), followed by a gradual decline. Calcium fluxes are closely linked to mitochondrial and inflammatory signaling, and transient elevations of calcium may participate in the regulation of pain mediators, while the absence of sustained overload prevents the activation of cytotoxic pathways [19]. Maintaining calcium at non-injurious levels is crucial, as persistent overloads are associated with exacerbated nociception and neuroinflammation [20].

ROS levels increased significantly after irradiation, particularly within the first hour. This increase likely represents a transient oxidative burst, a well-documented mechanism of photobiomodulation (low intensity) [1,8,21]. ROS levels were significantly elevated across all protocols, except for Protocol B at 24 h. However, these increases did not exceed thresholds typically associated with oxidative damage, and no evidence of cytotoxicity was observed in parallel assays (cell viability, apoptosis, and dsDNA release). This pattern suggests that the ROS elevations were transient and more likely reflected redox signaling than injurious oxidative stress. Such controlled increases in ROS have been described as triggers of protective and anti-inflammatory pathways [1,14,22], supporting the interpretation of adaptive rather than deleterious responses.

MMP analysis revealed a marked transient hyperpolarization at 30 min in all protocols, indicating an early increase in mitochondrial activity. At 24 h, values tended to return toward baseline, with the most pronounced decline observed in Protocol B (not statistically different from control), whereas Protocols A and C remained significantly elevated compared to control. Decreases or increases in MMP are associated with different cellular mechanisms, including regulation of the cell cycle [23], apoptotic control [24], energy production [25], and oxidative stress [26]. In their recent review, Begum and Shen [25] highlighted the critical role of the mitochondrial calcium uniporter in regulating MMP. In this context, and considering the patterns observed for Ca^2+^ influx and ROS levels, both of which showed similar early increases followed by a decline, it is plausible that these fluctuations act in a coordinated manner to modulate MMP. This set of signals suggests a bioenergetic adaptive mechanism, where transient elevations in Ca^2+^ and ROS trigger mitochondrial adjustments that contribute, at least in part, to the delayed effects on ATP production. Importantly, no evidence of cytotoxicity was detected, supporting the interpretation that these MMP fluctuations reflect adaptive mitochondrial responses rather than dysfunction.

Regarding apoptosis, Protocols A and B showed a reduction in apoptotic cells at various time points, which may be related to the preservation of cell integrity and prevention of pro-inflammatory mediator release that would otherwise intensify nociception. In contrast, Protocol C induced a late increase in apoptosis, suggesting that, under specific conditions of higher energy density, activation of pro-apoptotic pathways may occur. Nevertheless, the absence of significant extracellular dsDNA release confirms that no massive cell lysis occurred, reinforcing the overall biocompatibility of the protocols used.

The selective increase in NO in Protocol A after 24 h is also noteworthy. NO, at moderate levels, can modulate neuronal and glial activity, regulate nociceptive synapses, and contribute to neuroprotective and anti-inflammatory effects [20,22]. Previous studies with defocused high-power laser have shown that NO modulation is associated with reduced pro-inflammatory cytokines and rebalancing of the redox state during repair processes [27].

Taken together, our results demonstrate that high-power laser therapy, applied with controlled parameters, modulated several aspects of microglial physiology in a protocol- and time-dependent manner. Protocol A was characterized by reduced ATP production, modulation of calcium levels, moderate NO increase, and absence of late apoptosis. Protocols B and C, in contrast, were associated with more pronounced stimulation of bioenergetic and mitochondrial activity, reflected in significant increases in ATP and MMP at 24 h. These findings reinforce the notion that variations in irradiance and exposure time, even under identical total energy, can lead to distinct cellular responses in BV-2 microglial cells.

These findings demonstrate similarities between PBM and high-power laser therapy and further suggest that high-power laser therapy also exhibits biphasic effects dependent on irradiance (power density) and energy density. In our study, despite identical total energy (657 J), the protocols differed in irradiance and exposure duration (A: 584 mW/cm^2^/225 s; B: 973 mW/cm^2^/135 s; C: 1460 mW/cm^2^/90 s), which likely contributed to the distinct temporal cellular responses observed. The safety and responsiveness of microglial cells to high-power laser therapy (class IV) observed in this study support its potential use as a modulatory tool in inflammatory and nociceptive models. Future studies should confirm these effects in vivo, evaluate the impact on the release of pro- and anti-nociceptive mediators, and further investigate wavelength-specific contributions using monochromatic sources.

As in all scientific research, this study presents limitations. High-power laser therapy can induce thermal effects, and although we standardized the distance between the laser emitter and culture plates to simulate in vivo attenuation, no thermal monitoring was performed during irradiation, making it difficult to distinguish between photobiological and potential thermal effects. Furthermore, the device used in our study delivers multiple wavelengths simultaneously, which may have enhanced the overall cellular response but limited our ability to attribute specific results to individual wavelengths.

Although this study was conducted in an in vitro model, the findings provide valuable insights into the cellular safety profile of high-power laser therapy and its potential to modulate microglial physiology. Given the central role of microglia in the conduction and amplification of nociceptive signaling within the central nervous system, these results highlight the therapeutic potential of this approach. Nevertheless, further investigations in animal models and clinical settings are essential to confirm whether the cellular effects observed here can be translated into meaningful therapeutic outcomes, particularly in the context of pain modulation and control.

## 5. Conclusions

This study demonstrates that high-power laser therapy (class IV), when applied under controlled and optimized parameters, does not induce cytotoxicity in BV-2 microglial cells and is capable of modulating essential cellular processes, including mitochondrial function, redox balance, and apoptosis. The effects were both protocol- and time-dependent, with Protocols B and C promoting increases in ATP production and mitochondrial membrane potential, while Protocol A was distinguished by reduced ATP levels, nitric oxide modulation, and anti-apoptotic effects. Importantly, no evidence of membrane rupture or significant DNA release was detected in any protocol, supporting the overall biocompatibility of high-power laser therapy under the conditions tested.

These findings reinforce the safety profile of high-power laser therapy and suggest its potential to modulate microglial function in contexts such as neuroinflammation, pain control, and neurodegenerative diseases. However, further studies are required to elucidate the underlying molecular mechanisms, evaluate long-term effects, and determine its impact under pathological conditions, particularly in vivo models of acute and chronic pain.

## Figures and Tables

**Table 1:** Laser Parameters.

Protocols (Parameter or Method)	A (Unit)	B (Unit)	C (Unit)
Number of lasers	4	4	4
Wavelength (nm)	650/810/915/980	650/810/915/980	650/810/915/980
Frequency (Hz)	CW	CW	CW
Peak power (W)	0.2/1.0/1.1/1.5	0.2/2.0/2.2/3.0	0.2/4.0/4.4/6.0
Average mean optical output (mW)	2920	4866.7	7300
Power density (mW/cm^2^)	584	973.3	1460
Energy density (J/cm^2^)	131.4	131.4	131.4
Dose (J)	657	657	657
Spot size of laser (cm^2^)	5	5	5
Irradiation time (s)	225	135	90
Distance from the plate (cm)	34	33	33.1

nm (nanometer); Hz (hertz); W (watts); mW (microwatts); mW/cm^2^; (microwatt per centimeter square); J (joules); J/cm^2^ (joules per square centimeter); cm^2^ (square centimeter); s (seconds).

## Data Availability

The original contributions presented in this study are included in the article and Supplementary Materials. Further inquiries can be directed to the corresponding author.

## Supplementary Materials

The following supporting information can be downloaded at: https://www.mdpi.com/article/10.3390/antiox14101243/s1, Table S1: Outcomes.
