# Supplementary material for: High-Power Laser Therapy Modulates Mitochondrial Function and Redox Balance Without Cytotoxicity: An In Vitro Study in BV-2 Microglial Cells
Source: Antioxidants (Basel). 2025 Oct 16;14(10):1243. doi: 10.3390/antiox14101243 (PMC12562247; doi:10.3390/antiox14101243)
Supplement: Supplementary file 1 [file antioxidants-14-01243-s001.zip › antioxidants-3856199-supplementary.pdf]

Supplementary Table S1. Outcomes

		Control	Protocol A	Protocol B	Protocol C
**Cell Viability (Absorbance - Mean±SD)**	5 min	0.75±0.01	0.43±0.02	0.75±0.04	0.44±0.03
	10min		0.69±0.02	0.68±0.02	0.66±0.02
	30min		0.81±0.06	0.96±0.05	0.65±0.03
	1h		0.85±0.06	0.75±0.04	0.96±0.04
	3hs		0.93±0.01	0.77±0.04	0.78±0.01
	24hs		0.83±0.04	1.13±0.05	1.02±0.05
**ATP Production (RLU -Mean±SD)**	5 min	380,000±5,595.32	298,368.33±8,169.71	354,636.66±5,595.32	326,743.33±14,791.21
	30min		354,637.00±6,129.36	391,391.67±6,347.27	418,201.66±19,806.20
	1h		326,743.00±16,202.96	394,570.00±15,757.78	383,151.66±22,659.82
	3hs		300,073.00±19,238.47	322,775.00±17,796.52	408,495.00±21,714.76
	24hs		380,695.00±7,867.59	463,811.67±32,470.03	908,090.00±19,095.81
**Calcium Levels (% AFU - Mean±SD)**	5 min	168,065±23.94	232.77±14.46	440.23±10.22	370.00±41.01
	30min		421.23±8.82	374.27±21.59	541.64±16.46
	1h		377.89±32.67	262.09±16.83	329.22±42.11
	3hs		321.12±43.66	261.97±5.61	337.67±6.60

	24hs		266.58±48.66	128.77±23.72	157.50±4.94
**Reactive Oxygen Species (% AFU - Mean±SD)**	5 min		31.50±2.26	26.57±3.57	27.36±2.17
	30min		37.88±4.63	28.26±1.07	38.00±0.98
	1h	7.75±0.52	27.47±2.44	30.75±1.48	29.60±5.23
	3hs		27.20±2.70	30.47±3.49	29.00±3.25
	24hs		14.31±1.00	12.39±2.55	24.66±1.89
**Mitochondrial Membrane Potential (% AFU - Mean±SD)**	5 min		292.58±7.59	248.50±27.57	254.00±32.52
	30min		369.86±5.86	316.47±17.71	309.21±27.98
	1h	108.88±25.61	287.35±4.35	295.83±5.89	291.70±13.13
	3hs		268.58±36.41	185.50±6.36	245.40±7.63
	24hs		230.71±24.72	149.21±8.18	204.43±3.62
**Apoptosis (% - Mean±SD)**	5 min		3.18±0.10	3.48±0.18	2.00±0.23
	30min		3.65±0.05	2.46±1.05	7.36±1.00
	1h	6.90±0.15	3.54±0.92	3.34±1.12	8.31±3.23
	3hs		7.15±0.57	6.45±0.10	7.82±1.43
	24hs		4.72±0.54	2.52±0.07	17.19±2.30
**dsDNA Release (A.U. -Mean±SD)**	5 min	1.08±0.01	0.99±0.11	1.13±0.02	0.98±0.07
	30min		1.00±0.12	1.08±0.06	1.04±0.08

	1h		1.00±0.10	1.07±0.05	1.03±0.12
	3hs		0.98±0.06	1.07±0.06	1.01±0.11
	24hs		0.98±0.09	1.12±0.06	1.01±0.10
**Nitric Oxide Production (Absorbance - Mean±SD)**	5 min		0.065±0.004	0.065±0.004	0.064±0.004
	30min		0.065±0.003	0.061±0.004	0.064±0.003
	1h	0.06±0.001	0.065±0.005	0.065±0.006	0.064±0.005
	3hs		0.066±0.005	0.065±0.006	0.063±0.005
	24hs		0.077±0.002	0.069±0.005	0.070±0.005

Legend: SD – Standard Deviation; RLU - Relative Luminescence Units; AFU - Arbitrary Fluorescence Units; A.U, - Adenine and Uracil; min-minutes; h-hour; hs- hours;
